# Association of the Great East Japan Earthquake and the Daiichi Nuclear Disaster in Fukushima City, Japan, With Birth Rates

**DOI:** 10.1001/jamanetworkopen.2018.7455

**Published:** 2019-01-25

**Authors:** Noriaki Kurita

**Affiliations:** 1 Department of Innovative Research and Education for Clinicians and Trainees, Fukushima Medical University Hospital, Fukushima, Japan; 2 Center for Innovative Research for Communities and Clinical Excellence, Fukushima Medical University, Fukushima, Japan

## Abstract

**Importance:**

The association of the Great East Japan Earthquake and the subsequent Fukushima Daiichi Nuclear Power Plant disaster of March 11 and 12, 2011, in Fukushima, Japan, with birth rates has not been examined appropriately in the existing literature.

**Objective:**

To assess the midterm and long-term associations of the Great East Japan Earthquake and the Fukushima Daiichi Nuclear Power Plant disaster with birth rates.

**Design, Setting, and Participants:**

Cohort study in which interrupted time series analyses were used to assess monthly changes in birth rates among residents of Fukushima City, Japan, from March 1, 2011, to December 31, 2017, relative to projected birth rates without the disaster based on predisaster trends. Birth rates from January 1, 2007, to December 31, 2017, in Fukushima City were determined using information from the Fukushima City government office.

**Exposure:**

The Great East Japan Earthquake and the Fukushima Daiichi Nuclear Power Plant disaster, expressed via 5 potential models of the association with birth rate: level change, level and slope changes, temporal level change, and temporal level change with 1 or 2 slope change(s).

**Main Outcomes and Measures:**

Birth rate, calculated from monthly data on the number of births and total population.

**Results:**

The mean birth rate before the Great East Japan Earthquake and the Fukushima Daiichi Nuclear Power Plant disaster was 69.8 per 100 000 people per month; after the disaster, the mean birth rate was 61.9 per 100 000 people per month. Compared with birth rates before the Great East Japan Earthquake and the Fukushima Daiichi Nuclear Power Plant disaster, there was an estimated 10% reduction in monthly birth rates in Fukushima City (rate ratio, 0.90; 95% CI, 0.86-0.93) in the first 2 years after the disaster. After that, the birth rate trend was similar to the predisaster trend. The predisaster trend suggested a continuous decrease in birth rate (rate ratio for 1 year, 0.98; 95% CI, 0.98-0.99). This gap model was optimal and parsimonious compared with others. A similar association was found when trimonthly averaged data were analyzed.

**Conclusions and Relevance:**

The Great East Japan Earthquake and the Fukushima Daiichi Nuclear Power Plant disaster were followed by significant reductions in birth rates for 2 years. There was insufficient evidence to indicate that the trend in the 3 to 7 years after the disaster differed from the predisaster trends. The recovery from the reductions in the birth rate may be indicative of the rebuilding efforts. The continuing long-term decrease in birth rates observed before the Great East Japan Earthquake and the Fukushima Daiichi Nuclear Power Plant disaster suggests that continuing measures to support birth planning should be considered at the administrative level.

## Key Points

**Question:**

What was the long-term association of the Great East Japan Earthquake and the Fukushima Daiichi Nuclear Power Plant disaster with birth rates in Fukushima City, Japan?

**Findings:**

In this cohort study using interrupted time series analyses, the birth rate decreased by 10% in the 2 years after the Fukushima Daiichi Nuclear Power Plant disaster. However, there was insufficient evidence to indicate that the trend in the 3 to 7 years after the disaster differed from the predisaster trend.

**Meaning:**

After the Great East Japan Earthquake and the Fukushima Daiichi Nuclear Power Plant disaster, birth rates in Fukushima City decreased temporarily and then returned to predisaster levels after 2 years.

## Teaser

This cohort study uses interrupted time series analyses to examine the midterm and long-term associations of the Great East Japan Earthquake and the Fukushima Daiichi Nuclear Power Plant disaster with birth rates.

## Introduction

The Great East Japan Earthquake and the subsequent tsunami that occurred on March 11, 2011, led to the Fukushima Daiichi Nuclear Power Plant (FDNPP) disaster. This disaster forced those who lived within 20 km of the FDNPP to evacuate, according to the Disaster Countermeasures Basic Act. The disaster increased stress levels among women of childbearing age, making them especially anxious about the potential effects of radioactive material released from the FDNPP on conception and fetuses. Previous literature has addressed the issue of stress associated with the disaster, with some studies reporting the elevated incidence of cardiovascular diseases, such as congestive heart failure^1^ and myocardial infarction,^2^ within 2 years after the disaster. On the other hand, to our knowledge, there has been very little work describing birth rates in the Fukushima Prefecture both before and after the disaster.

One previous study showed that the number of births decreased in the 13 prefectures affected by the Great East Japan Earthquake and the FDNPP disaster (including Fukushima prefecture) between December 2011 and June 2012.^3^ On the other hand, mass media reported that, while there was a slight recovery in the number of births in Fukushima Prefecture 2 years after the disaster (in 2013), the numbers decreased again in 2016 based on vital statistics in the prefecture.^4,5^ However, whether or how the trend in birth rates in the Fukushima Prefecture changed after the disaster has not been examined thoroughly in the extant literature. In addition, the trend after the disaster has not been considered both in the context of disaster and in the context of decreasing birth rates in Japan.

Fukushima City (hereafter simply referred to as Fukushima) is the capital of the prefecture, located in the northern part of the prefecture. It faces a decreasing birth rate, although it is approximately 60 km away from the FDNPP and was exempt from forced evacuation. This study analyzes the long-term associations between the Great East Japan Earthquake and the FDNPP disaster and the birth rates in Fukushima. Understanding these associations may help clarify how local governments can face similar disasters in the future.

## Methods

This study used data on birth numbers and the population in Fukushima from January 1, 2007, to December 31, 2017, to examine the associations between the FDNPP disaster and birth rates. Data were extracted from available data from the Fukushima City government.^6^ This study is exempt from the evaluation of an institutional review board, because Japanese ethical guidelines do not mandate ethical review for studies analyzing publicly available data. In addition, because this study used anonymized aggregated data, informed consent was not mandatory according to the ethical guidelines. Although this was an epidemiologic study, its interrupted time series analysis study design could not be categorized as a cohort, case-control, or cross-sectional study. Thus, no reporting guideline was applicable.

The main outcome in this study is birth rate, defined by the number of births registered in Fukushima per month divided by the city’s population at the beginning of the same month. The birth number is counted based on data on birth registration submitted to local governments in Fukushima. Birth registration is mandatory for all live births in Japan, and requires a date of birth and an address clarification for resident registration. If the address for resident registration is in Fukushima, then the case is included for the outcome. Month of birth is determined by the date written on the birth registration.

All statistical analyses were conducted using Stata/SE, version 15 (StataCorp). Monthly birth rates during the study period were plotted and the mean birth rates before and after the disaster were calculated. Interrupted time series analysis was used to estimate changes in the level or trends of birth rates by fitting a series of Poisson regression models with the overdispersion parameter estimated using the Pearson χ^2^ statistic, divided by the residual *df*. The logarithm of monthly population numbers in Fukushima was used as an offset parameter. Five models of the association between the Great East Japan Earthquake and the FDNPP disaster and birth rates were used: (1) a change in the level, (2) a change in both the level and the trend, (3) temporal level change (ie, gap), (4) gap plus a change in the trend after the gap period, and (5) gap plus a change in the trends after the disaster and after the gap period (eTable 1 in the Supplement).^7^ Choice of those models, including at least a change in the level at the time of the disaster, was based on knowledge of the disaster and its expected effects on the outcome. A 2-year window was assumed for 2 reasons. First, I assumed that the birth rate would recover in March 2013, considering the timing of a report by the Exploratory Committee on Fukushima Health Management in June 2012, which stated that “rates of abortion and miscarriage did not differ between the pre- and post-disaster periods.”^8^^(p15-17)^ Second, since the gestation period for humans is 9 months after conception, the period extended to March 2013. Furthermore, 2 additional models corresponding to each outcome model were fitted, to specify the seasonal component in 2 ways. In the seasonality-adjusted models, the following variables were added to the variables used for the original outcome model: (1) indicator variables corresponding to the calendar month or (2) sine and cosine pairs expressing the secular trends, namely, sin (2π × *t*/12), cos (2π × *t*/12), sin (4π × *t*/12), and cos (4π × *t*/12), where *t* indicates secular 12 months. To compare the impact models, both the Akaike information criterion and the Bayesian information criterion were reported. From the fitted outcome model, the predicted birth rate trend before the disaster, the predicted birth rate trend after the disaster, and the projected trend of the predisaster period were plotted. In addition, rate ratios (RRs) and their 95% CIs were estimated from the outcome models. Sensitivity analyses were also conducted. All the analyses were repeated after using the monthly birth rates to calculate a mean quarterly birth rate. One exception was seasonal adjustment, which was accomplished by adjusting the quarter as an indicator variable. All *P* values were from 2-sided tests and results were deemed statistically significant at *P* < .05.

## Results

The plot of birth rates is shown in the Figure. The mean birth rate before the Great East Japan Earthquake and the FDNPP disaster was 69.8 per 100 000 people per month and after the disaster was 61.9 per 100 000 people per month. After the Great East Japan Earthquake and the FDNPP disaster, the birth rate apparently decreased during the first 2 years, that is, from March 2011 to February 2013 (mean birth rate during the first 2 years after the disaster, 59.5 per 100 000 people per month), and increased again from March 2013 to December 2017 (mean birth rate during 3-7 years after the disaster, 62.9 per 100 000 people per month).

**Figure.**
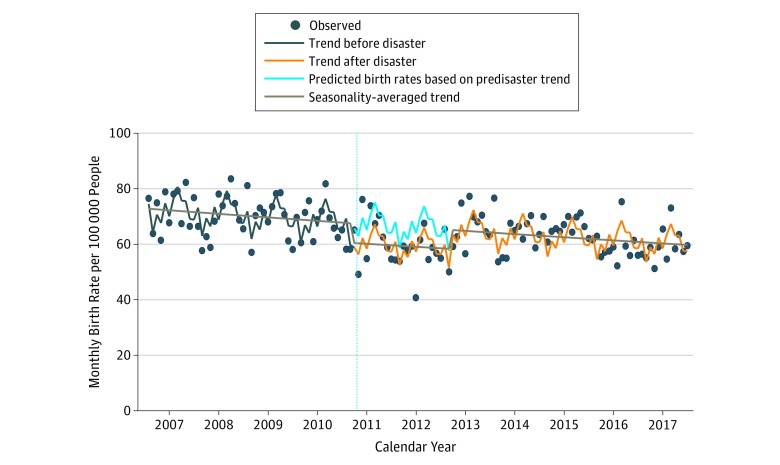
Trend in Birth Rates Estimated From Interrupted Time Series Analysis The trend in birth rates was estimated using the Poisson regression model, including the temporal level change (gap) and the indicator variable of the calendar month. The rate ratio during the 2 years after the Fukushima Daiichi Nuclear Power Plant disaster was 0.90 (95% CI, 0.86-0.93). The dots indicate observed birth rates. The vertical line indicates the time of the Fukushima Daiichi Nuclear Power Plant disaster between February and March 2011.

Values of the Akaike information criterion and the Bayesian information criterion calculated from a series of time-interrupted series analyses are presented in the Table. Based on the values of the Akaike information criterion and the Bayesian information criterion, the model including the gap and the seasonal adjustment with the indicator variable of the calendar month has the lowest values, whereas the model including the gap plus change in trends 2 years after the disaster, with the indicator variable of the calendar month, has the second lowest values. However, none of the changes in the trend component that were added to the gap component were statistically significant. Taken together with the values in the Table, models including gap and seasonal adjustments are optimal and parsimonious. Birth rates estimated from the models are presented in the Figure and eFigure 1 in the Supplement. The data from before the disaster suggest a long-term trend of declining birth rates (RR for 1 year, 0.98; 95% CI, 0.98-0.99). In the 2 years immediately after the disaster, the birth rate decreased (RR, 0.90; 95% CI, 0.86-0.93). However, after 2 years, there was insufficient evidence to support that the trend in birth rate was different from the trend estimated from the predisaster period.

**Table.** Values of the Akaike Information Criterion (AIC) and the Bayesian Information Criterion (BIC) Calculated From Models for Interrupted Time Series Analysis^a^

Model	Parameters, No.	AIC	BIC
Change in level	3	1240.3	1249.0
Change in level plus seasonality adjustment (indicator)	14	1164.8	1205.2
Change in level plus seasonality adjustment (sine and cosine pairs)	7	1191.5	1211.7
Change in level and trend	4	1237.5	1249.0
Change in level and trend plus seasonality adjustment (indicator)	15	1161.8	1205.0
Change in level and trend plus seasonality adjustment (sine and cosine pairs)	8	1188.1	1211.1
Temporal level change (gap)	3	1210.9	1219.6
Temporal level change (gap) plus seasonality adjustment (indicator)	14	1135.5	1175.9
Temporal level change (gap) plus seasonality adjustment (sine and cosine pairs)	7	1161.1	1181.3
Gap plus change in trend at 2 y after the disaster	4	1212.5	1224.0
Gap plus change in trend at 2 y after the disaster plus seasonality adjustment (indicator)	15	1137.0	1180.3
Gap plus change in trend at 2 y after the disaster plus seasonality adjustment (sine and cosine pairs)	8	1162.6	1185.7
Gap plus changes in trends at the disaster and 2 y after the disaster	5	1214.5	1228.9
Gap plus changes in trends at the disaster and 2 y after the disaster plus seasonality adjustment (indicator)	16	1138.9	1185.0
Gap plus changes in trends at the disaster and 2 y after the disaster plus seasonality adjustment (sine and cosine pairs)	9	1164.5	1190.4

^a^ Poisson regression models with adjustment of scale parameter were used to estimate birth rate. Change in level modeled intercept change after March 2011. Change in level and trend modeled intercept and slope changes after March 2011. Temporal level change (ie, gap) modeled intercept change for the 2 years after March 2011. Gap plus change in trend modeled gap plus slope change 2 years after March 2011. Gap plus changes in trends modeled gap plus slope changes after March 2011 and 2 years after March 2011. Seasonality adjustment was applied by including indicator variables of the calendar month (“indicator”) or sine and cosine pairs (sin [2π × *t*/12], cos [2π × *t*/12], sin [4π × *t*/12], and cos [4π × *t*/12], where *t* indicates secular 12 months). None of the changes in the trend component added to the gap component were statistically significant. Taken together with this table, models including temporal level changes with seasonality adjustment are optimal and parsimonious.

A series of sensitivity analyses corroborated the results presented above (eTable 2 and eFigure 2 in the Supplement). The sensitivity analyses also supported the gap model with a seasonal adjustment as the optimal approach. The magnitude of the RR during the 2 years immediately after the disaster and the magnitude of the RR in the long-term trend of decreasing birth rates in the sensitivity analysis were similar to those presented in the Figure.

## Discussion

The decreased number of births from December 2011 to June 2012 in the 13 prefectures affected by the Great East Japan Earthquake and the FDNPP disaster (including Fukushima prefecture) was reported by a previous study.^3^ However, as its authors acknowledged clearly, only a short period (7 months) was examined. In contrast, the present study examined a period of 11 years and identified long-term trends in birth rates using an interrupted time series design.

This study has several implications for governments of and residents in local communities that have nuclear power plants but are distant from them. First, timely measurement of the area’s radioactive materials in the environment (ie, the air, soil, food, and water) is essential for reasonable decision making, as is providing the results and accurate probabilities of reproductive effects to the residents. The decrease in the birth rate immediately after the FDNPP disaster can be explained by the decision of pregnant women to relocate further away from the FDNPP than Fukushima, for fear of potentially hazardous effects from radiation. The regular measurement of the radioactive materials enabled the estimation of a cumulative dose and allowed the governments to reasonably decide that Fukushima was exempt from being a forced evacuation zone. In addition, simultaneous measurement of radioactive materials in food and water and timely interventions such as regulating the water supply corporation and reasonable instructions for the residents on whether or not they could use tap water, such as the instruction provided jointly by the Japanese Pediatric Society and other related societies,^9^ were helpful for pregnant women to minimize radiation exposure for their fetus and near future infants. Furthermore, proactive announcements of the operating status of perinatal care facilities available for pregnant women would also be helpful. The more immediate and accurate these governmental responses are, the more reassured and confident that pregnant women will be about remaining in Fukushima.

Second, continuous monitoring and timely reporting of radiation exposure of the residents^10^ and perinatal outcomes were also helpful for women of childbearing age. The proceedings of the Seventh Exploratory Committee on Fukushima Health Management held in June 2012 revealed that rates of abortion and miscarriage did not differ between the predisaster and postdisaster periods.^8^ The recovery after the sustained decrease in birth rates observed in March 2013, a period of 9 months after the committee met, may be partially explained by the recovery from the lack of or decrease in pregnancies since the report of the committee, considering the approximately 9-month gestation period. Thus, immediate reporting of perinatal outcomes may contribute to shortening the duration of the decrease in birth rates.

Third, continuing measures to support birth planning should be considered at the local government level in the long term, as these findings support the notion that the decrease in birth rates in recent years can be explained by a long-term trend of decreasing birth rates observed before the disaster, owing to an aging population, a decrease in the rate of marriage, an increase in mean age at marriage, and/or a decrease in desire for childbearing. Thus, the local government may have to address these problems. Previous research suggests that an increase in the availability of childcare was associated with an increase in the fertility rate among young adult women living in certain regions of Japan.^11^

### Limitations

This study has some limitations. First, it potentially underestimates birth rates several years after the FDNPP disaster. If some mothers gave birth after relocation to other municipalities but left their resident registration unchanged, they might write “Fukushima” on their birth registration documents. Further investigation may be warranted regarding whether this practice was common or not. Second, this study was not able to analyze the total fertility rate because of the lack of detailed data. Compared with the birth rate, the total fertility rate may not be affected by an aging population.

## Conclusions

The FDNPP disaster was associated with a 2-year decrease in birth rates in the nearby city of Fukushima. The recovery from this decrease in birth rates may be indicative of the rebuilding efforts. After recovery, the city still seems to be facing a decreasing trend without sufficient evidence to indicate a difference from the predisaster trend. Further studies on birth rates at the prefectural level are warranted to explore how the association between the FDNPP disaster and birth rates differs by residents’ distance from the FDNPP.

## Notes

Supplement. **eTable 1.** List of the Impact Models and Their Parameters Used in This Study **eTable 2.** Values of Akaike Information Criterion and Bayesian Information Criterion Calculated From Impact Models for Interrupted Time Series Analysis Using Trimonthly-Averaged Data **eFigure 1.** Trend in Birth Rates Estimated From Interrupted Time Series Analysis **eFigure 2.** Trend in Birth Rates Estimated From Interrupted Time Series Analysis Using Trimonthly-Averaged Data
