# Error in Sentence Wording

**DOI:** 10.1001/jamanetworkopen.2019.0588

**Published:** 2019-02-22

In the Original Investigation titled “Association of the Great East Japan Earthquake and the Daiichi Nuclear Disaster in Fukushima City, Japan, With Birth Rates,”^1^ published January 25, 2019, there was a wording error in the fourth sentence of the Limitations section, which should have read as follows: “Further investigation may be warranted regarding whether this practice was common or not.” This article was corrected online.^1^
