# Supplementary material for: Association of the Great East Japan Earthquake and the Daiichi Nuclear Disaster in Fukushima City, Japan, With Birth Rates
Source: JAMA Netw Open. 2019 Jan 25;2(1):e187455. doi: 10.1001/jamanetworkopen.2018.7455 (PMC6484541; doi:10.1001/jamanetworkopen.2018.7455)
Supplement: Supplement. — eTable 1. List of the Impact Models and Their Parameters Used in This Study eTable 2. Values of Akaike Information Criterion and Bayesian Information Criterion Calculated From Impact Models for Interrupted Time Series Analysis Using Trimonthly-Averaged Data eFigure 1. Trend in Birth Rates Estimated From Interrupted Time Series Analysis eFigure 2. Trend in Birth Rates Estimated From Interrupted Time Series Analysis Using Trimonthly-Averaged Data [file jamanetwopen-2-e187455-s001.pdf]

Supplementary Online Content

Kurita N. Association of the Great East Japan Earthquake and the Daiichi nuclear disaster in Fukushima City, Japan, with birth rates. *JAMA Netw Open.* 2019;2(1):e187455. doi:10.1001/jamanetworkopen.2018.7455

eTable 1. List of the Impact Models and Their Parameters Used in This Study

eTable 2. Values of Akaike Information Criterion and Bayesian Information Criterion Calculated From Impact Models for Interrupted Time Series Analysis Using Trimonthly-Averaged Data

eFigure 1. Trend in Birth Rates Estimated From Interrupted Time Series Analysis

eFigure 2. Trend in Birth Rates Estimated From Interrupted Time Series Analysis Using Trimonthly-Averaged Data

This supplementary material has been provided by the authors to give readers additional information about their work.

© 2019 Kurita N. *JAMA Network Open.*

eTable 1. List of the Impact Models and Their Parameters Used in This Study

Impact model	Parameters in the impact model
**Change in level**<br><br>$\boldsymbol{Birth\ rate_t = exp(\beta_0 + \beta_1 T + \beta_2 D_t)}$<br><br>T: the time in months at time T = *t* since the start of the observation; $\boldsymbol{D_t}$: a dummy variable indicating the pre-disaster period ($\boldsymbol{D_t}$= 0 if *t* ≤ February 2011) or the post-disaster period ($\boldsymbol{D_t}$= 1 if *t* ≥ March 2011); $\boldsymbol{Birth\ rate_t}$: the monthly birth rate per people at T = *t*.	Here, β_0 represents the intercept at T = 0, β_1 is interpreted as the change in birth rate associated with a one-month increase (representing the underlying pre-disaster trend), β_2 is the level change following the disaster (*t* ≥ March 2011).
**Change in level and trend**<br><br>$\boldsymbol{Birth\ rate_t = exp(\beta_0 + \beta_1 T + \beta_2 D_t + \beta_3 T^* D_t)}$<br><br>T: the time in months at time T = *t* since the start of the observation; $\boldsymbol{D_t}$: a dummy variable indicating the pre-disaster period ($\boldsymbol{D_t}$= 0 if *t* ≤ February 2011) or the post-disaster period ($\boldsymbol{D_t}$= 1 if *t* ≥ March 2011); T*: the number of months after the disaster at time T = *t* (namely, *t* – February 2011); $\boldsymbol{Birth\ rate_t}$: the monthly birth rate per people at T = *t*.	Here, β_0 represents the intercept at T = 0, β_1 is interpreted as the change in birth rate associated with a one-month increase (representing the underlying pre-disaster trend), β_2 is the level change following the disaster (*t* ≥ March 2011), β_3 indicates the slope change following the disaster (representing the change in the trend, using the interaction between T* and D_t: $T^* D_t$). The sum of β_1 and β_3 is the post-disaster trend.
**Temporal level change (gap)**<br><br>$\boldsymbol{Birth\ rate_t = exp(\beta_0 + \beta_1 T + \beta_2^* D_t^*)}$<br><br>T: the time in months at time T = *t* since the start of the observation; $\boldsymbol{D_t^*}$: a dummy variable indicating the period during two years after the disaster ($\boldsymbol{D_t^*}$= 1 if March 2011 ≤ *t* ≤ February 2013) or not ($\boldsymbol{D_t^*}$= 0 if *t* ≤ February 2011 or t ≥ March 2013); $\boldsymbol{Birth\ rate_t}$: the monthly birth rate per people at T = *t*.	Here, β_0 represents the intercept at T = 0, β_1 is interpreted as the change in birth rate associated with a one-month increase (representing the underlying pre-

	disaster trend), β_2^* is the temporal level change during two years after the disaster (March 2011 ≤ t ≤ February 2013).
**Gap + change in trend at two years after the disaster**<br><br>$\boldsymbol{Birth\ rate_t = exp(\beta_0 + \beta_1 T + \beta_2^* D_t^* + \beta_3^* T^{**} D_t^{**})}$<br><br>T: the time in months at time T = t since the start of the observation; $\boldsymbol{D_t^*}$: a dummy variable indicating the period during two years after the disaster ($\boldsymbol{D_t^*}$= 1 if March 2011 ≤ t ≤ February 2013) or not ($\boldsymbol{D_t^*}$= 0 if t ≤ February 2011 or t ≥ March 2013) ; T**: the number of months since the two years after the disaster at time T = t (namely, t – February 2013); $\boldsymbol{D_t^{**}}$: a dummy variable indicating the period since two years after the disaster ($\boldsymbol{D_t^{**}}$= 1 if March 2013 ≤ t) or before ($\boldsymbol{D_t^{**}}$= 0 if t ≤ February 2013); $\boldsymbol{Birth\ rate_t}$: the monthly birth rate per people at T = t.	Here, β_0 represents the intercept at T = 0, β_1 is interpreted as the change in birth rate associated with a one-month increase (representing the underlying pre-disaster trend), β_2^* is the temporal level change during two years after the disaster (March 2011 ≤ t ≤ February 2013), β_3^* indicates the slope change since the two years after the disaster (representing the change in the trend, using the interaction between T** and D_t^{**} : $T^{**}D_t^{**}$). The sum of β_1 and β_3^* is the trend since the two years after the disaster.
**Gap + changes in trends at the disaster and two years after**<br><br>$\boldsymbol{Birth\ rate_t = exp(\beta_0 + \beta_1 T + \beta_2^* D_t^* + \beta_3 T^* D_t + \beta_3^* T^{**} D_t^{**})}$<br><br>T: the time in months at time T = t since the start of the observation; $\boldsymbol{D_t^*}$: a dummy variable indicating the period during two years after the disaster ($\boldsymbol{D_t^*}$= 1 if March 2011 ≤ t ≤ February 2013) or not ($\boldsymbol{D_t^*}$= 0 if t ≤ February 2011 or t ≥ March 2013) ; T*: the number of months after the disaster at time T = t (namely, t – February 2011) ; T**: the number of months since the two years after the disaster at time T = t (namely, t – February 2013); $\boldsymbol{D_t^{**}}$: a dummy variable indicating the period since two years after the disaster ($\boldsymbol{D_t^{**}}$= 1 if March 2013 ≤ t) or before ($\boldsymbol{D_t^{**}}$= 0 if t ≤ February 2013); $\boldsymbol{Birth\ rate_t}$: the monthly birth rate per people at T = t.	Here, β_0 represents the intercept at T = 0, β_1 is interpreted as the change in birth rate associated with a one-month increase (representing the underlying pre-disaster trend), β_2^* is the temporal level change during two years after the disaster (March 2011 ≤ t ≤ February 2013), β_3 indicates the slope change following the disaster (representing the change in the

© 2019 Kurita N. *JAMA Network Open.*

	trend, using the interaction between T* and D_t : T^*D_t), β_3^* indicates the slope change since the two years after the disaster (representing the change in the trend, using the interaction between T** and D_t^{**} : $T^{**}D_t^{**}$). The sum of β_1 and β_3 is the trend during two years after the disaster. The sum of β_1, β_3, and β_3^* is the trend since the two years after the disaster.

Seasonality adjustment was applied by including (1) the indicator variable of calendar month ($\beta_4 T_{feb} + \beta_5 T_{march} + \beta_6 T_{apr} + \beta_7 T_{may} + \beta_8 T_{jun} + \beta_9 T_{jul} + \beta_{10} T_{aug} + \beta_{11} T_{sep} + \beta_{12} T_{oct} + \beta_{13} T_{nov} + \beta_{14} T_{dec}$), where T_i indicates a dummy variable indicating calendar month *i* (e.g. if T = February 2011, then $T_{feb} = 1$, and $T_i = 0$ for *i* = month other than January and February), or (2) sine and cosine pairs [$\beta_4 \sin(2\pi \times t/12) + \beta_5 \cos(2\pi \times t/12) + \beta_6 \sin(4\pi \times t/12) + \beta_7 \cos(4\pi \times t/12)$], where *t* indicates secular 12 months].

© 2019 Kurita N. *JAMA Network Open.*

eFigure 1. Trend in Birth Rates Estimated From Interrupted Time Series Analysis

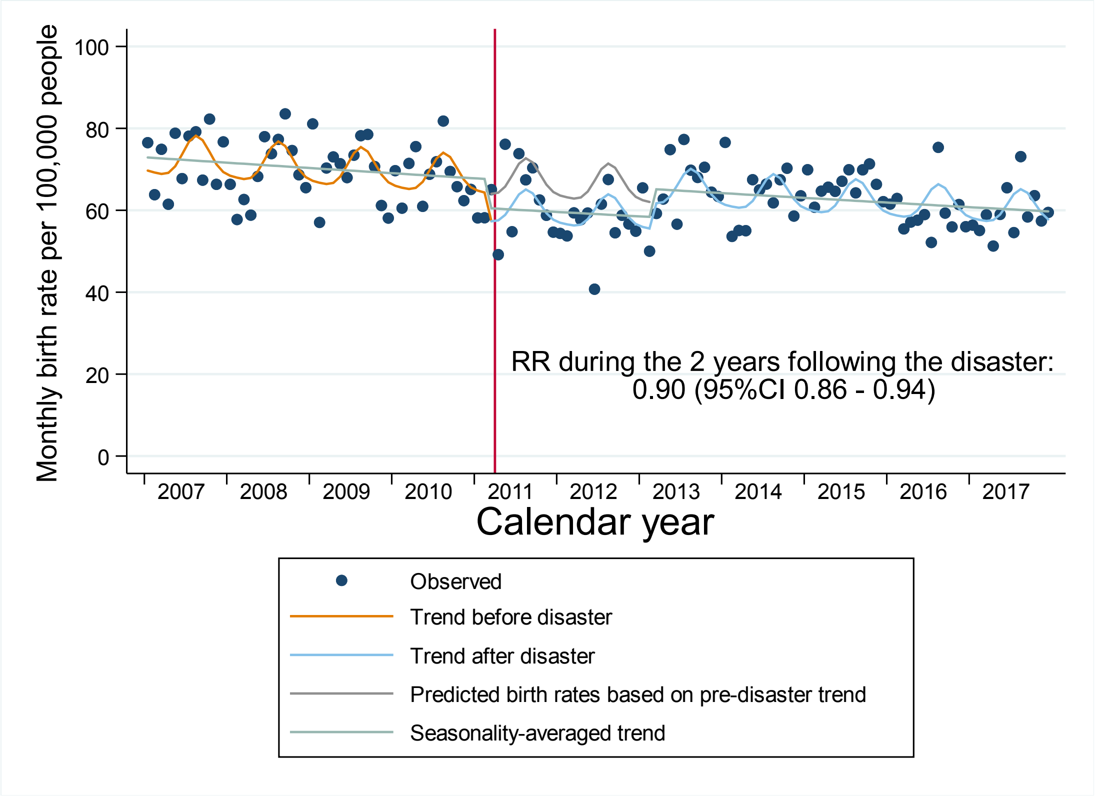

The trend in birth rates was estimated using the Poisson regression model, including temporal level change (gap) and sine and cosine pairs [sin(2π×*t*/12), cos(2π×*t*/12), sin(4π×*t*/12), and cos(4π×*t*/12)]. The dots indicate observed birth rates. The vertical line indicates the time of the Fukushima Daiichi Nuclear Power Plant disaster between February and March 2011. The trend before the disaster suggests a long-term trend of declining birth rate with seasonal change (rate ratio [RR] for 1 year: 0.98, 95% confidence interval [95%CI]: 0.98 - 0.99). Two years after the disaster, the birth rate seemingly dropped (RR during the 2 years: 0.90, 95%CI 0.86 - 0.94). RR: rate ratio, 95%CI: 95% confidence interval.

© 2019 Kurita N. *JAMA Network Open.*

eTable 2. Values of Akaike Information Criterion and Bayesian Information Criterion Calculated From Impact Models for Interrupted Time Series Analysis Using Trimonthly-Averaged Data

Lists of the impact model used in this study	N of parameter	AIC	BIC
**Change in level**	3	361.2	366.6
**Change in level + seasonality adjustment (indicator)**	6	356.5	367.3
**Change in level and trend**	4	361.9	369.1
**Change in level and trend + seasonality adjustment (indicator)**	7	356.8	369.5
**Temporal level change (gap)**	3	351.4	356.8
**Temporal level change (gap) + seasonality adjustment (indicator)**	6	346.7	357.6
**Gap + change in trend at 2 years after the disaster**	4	353.2	360.4
**Gap + change in trend at 2 years after the disaster + seasonality adjustment (indicator)**	7	348.7	361.3
**Gap + changes in trends at the disaster and 2 years after**	5	355.2	364.2
**Gap + changes in trends at the disaster and 2 years after + seasonality adjustment (indicator)**	8	350.7	365.1

Poisson regression models with adjustment of scale parameter were used to estimate trimonthly-averaged birth rate. Change in level modeled intercept change after March 2011. Change in level and trend modeled intercept and slope changes after March 2011. Temporal level change (i.e., gap) modeled intercept change for the two years after March 2011. Gap plus changes in trends modeled gap plus slope changes after March 2011 and two years after March 2011. Seasonality adjustment was applied by including the indicator variable of the quarter (“indicator”). None of the changes in the trend component that added to the gap component were statistically significant. Taken together with this table, the model including temporal level change with seasonality adjustment is optimal. AIC: Akaike Information Criterion. BIC: Bayesian Information Criterion.

© 2019 Kurita N. *JAMA Network Open.*

eFigure 2. Trend in Birth Rates Estimated From Interrupted Time Series Analysis Using Trimonthly-Averaged Data

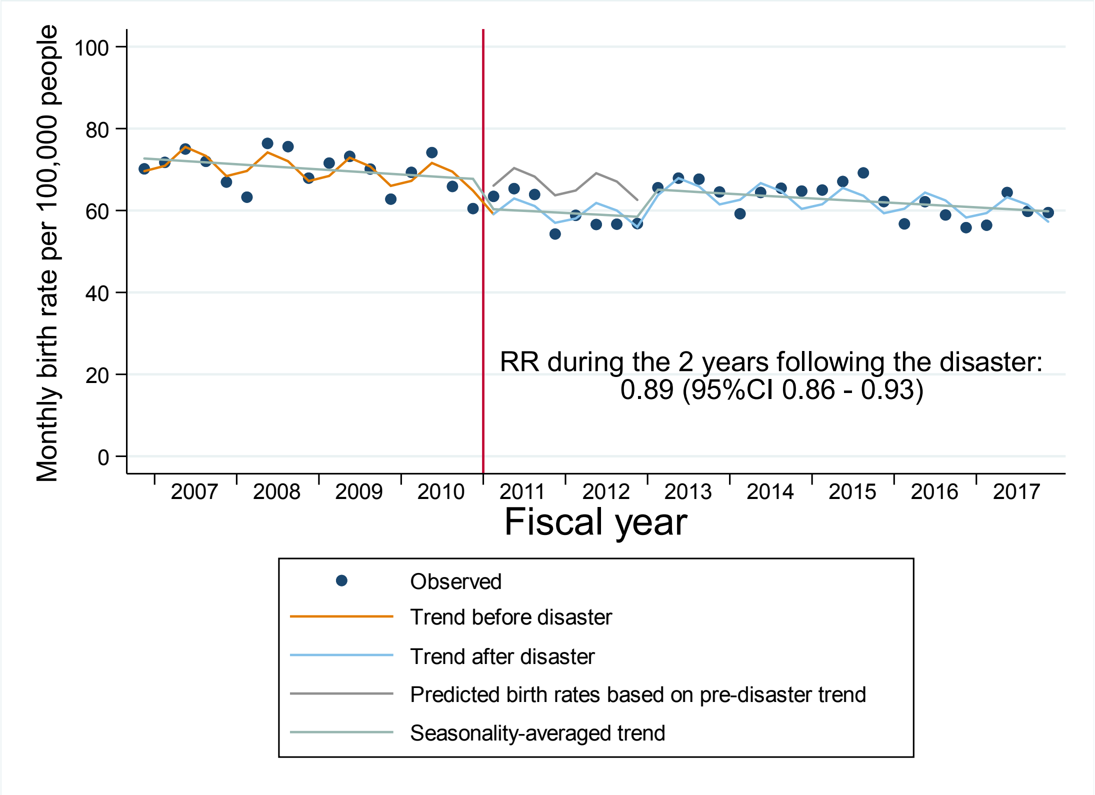

The trend in birth rates was estimated using the Poisson regression model, including temporal level change (gap) and the indicator variable of the quarter. The trend before the disaster suggests a long-term trend of declining birth rate with seasonal change (rate ratio [RR] for 1 year: 0.95, 95% confidence interval [95%CI]: 0.94 - 0.96). Two years after the disaster, birth rate seemingly dropped (RR during the 2 years: 0.89, 95%CI 0.86 - 0.93). The dots indicate observed birth rates. The vertical line indicates the time of the Fukushima Daiichi Nuclear Power Plant disaster between February and March 2011. RR: rate ratio, 95%CI: 95% confidence interval.

© 2019 Kurita N. *JAMA Network Open.*
